# Experimental study on tilting deformation and a new method for landslide prediction with retaining-wall locked segment

**DOI:** 10.1038/s41598-023-32477-9

**Published:** 2023-03-29

**Authors:** Han-dong Liu, Jing-jing Liu, Jia-xing Chen, Zhi-fei Guo, Lei Qiu

**Affiliations:** 1 Henan Key Laboratory of Geomechanics and Structural Engineering, North China University of Water Resources and Electric Power, Zhengzhou, 450046 China; 2 Powerchina Guiyang Engineering Corporation Ltd, Guizhou, 550081 China; 3 Powerchina Huadong Engineering Corporation Ltd, Hangzhou, 311122 China

**Keywords:** Civil engineering, Theory and computation, Natural hazards

## Abstract

The destruction of the locked-segment type landslide is often accompanied by the destruction of the locked segment with cumulative effects. Investigating the failure mode and instability mechanism of locked-segment type landslides is crucial. The study uses physical models to examine the evolution of locked-segment type landslides with retaining-walls. It utilizes a variety of instruments (tilt sensors, micro earth pressure sensors, pore water pressure sensors, strain gauges, and others) to conduct physical model tests of locked-segment type landslide with retaining-wall and to reveal the tilting deformation and evolution mechanism of retaining-wall locked landslide under the condition of rainfall. The results showed that the regularity of tilting rate, tilting acceleration, strain, and stress change in the retaining-wall locked segment is consistent with the landslide evolution process, indicating that tilting deformation can be used as the criterion of landslide instability and that the locked segment plays a vital role in controlling the landslide stability. The tertiary creep stages of tilting deformation are divided into initial, medium, and high tertiary creep stages using an improved angle tangent method. This establishes the failure criterion for locked-segment type landslides with tilting angles of 0.34°, 1.89°, and 4.38°. In addition, the tilting deformation curve of a locked-segment type landslide with a retaining-wall is utilized to predict the landslide instability by the reciprocal velocity method.

## Introduction

A landslide is a common global natural disaster^1^, and China is one of the countries with the most severe landslide disaster in the world. It characterizes by wide distribution, large quantity, and large scale, which often cause massive casualties and economic losses^2,3^. Huang^4^ and Xu et al.^5^ investigated typical large-scale catastrophic landslides in China and reported that the landslide stability was controlled by an internal unconnected locked segment that can withstand concentrated stress. As typical landslides, relevant scholars have extensively studied locked-segment type landslides^6–9^. The instability mechanism of various locked-segment type landslides varies. Pan^10^ classified locked-segment type landslides into five categories based on the types and formation conditions of the locked segment: cross-layer shear, direct bedding shear, homogeneous rock bridge, retaining-wall, and supporting arch. In addition, they proposed a prediction method for forming locked-segment type landslides. Liu et al.^11,12^ examined the geological conditions and induced factors of landslides in western Henan and concluded that the western region of locked-segment type landslide consists primarily of a direct shear layer, diagonally across the layer, and a retaining-wall. They also conducted large-scale physical model tests to investigate the disaster mechanism of locked landslides in western Henan. In the process of landslide evolution, when the stress experienced by the locked segment exceeds the shear strength, its internal accumulation of energy is suddenly released, and brittle failure occurs, resulting in the formation of high-speed and long-distance landslides, causing major geological disasters^13–15^. Therefore, the locked segment plays a controlling role in landslide deformation and stability. The study of the disaster mechanism of the locked-segment type landslides is vital for predicting and preventing the landslide disaster.

With the strengthening of national disaster prevention and control, landslide monitoring and warning gradually become an essential measure to reduce landslide disasters, and the landslide warning model and warning criterion are the key to investigating landslide warnings. Recently, scholars have explored disaster mechanisms, early identification, monitoring, and warning of geological disasters and have proposed numerous theoretical models and methods for landslide prediction^16–20^. Liu et al.^21^ developed the theory and model of landslide prediction and compiled the landslide deformation monitoring and prediction information system. Numerous studies have demonstrated that landslide disaster is closely related to rainfall capacity, intensity, and duration^22–27^. Glade^28^ analyzed the probability of landslide occurrence and proposed a threshold based on the established daily and previous rainfall models. Currently, landslide warning and prediction methods rely heavily on landslide displacement monitoring, but the traditional displacement monitoring equipment is limited due to its complex installation and maintenance. In addition, the emergence of remote sensing technology compensates for the deficiencies of traditional displacement monitoring methods, but it is challenging to apply this technology to landslide pre-slip monitoring because of its lack of precision and monitoring data period. Due to advancements in technology, the monitoring method for tilting landslide deformation has attracted the attention of numerous researchers due to its ease of installation and maintenance^29,30^.

The landslide's tilting deformation has become an essential monitoring and warning indicator in recent years. Studies have demonstrated that the slope angle increases as displacement rises in the landslide evolution process. Therefore, Iverson et al.^31^ regarded the landslide's tilting deformation as an essential criterion for landslide instability and conducted a series of studies based on this premise^32–34^. Xie et al.^35,36^ examined the tilting deformation characteristics through a series of model tests and field tests, revealed the linear relationship between tilting angle and displacement in the landslide sliding process, and proposed a new method to predict the occurrence of landslide based on tilting deformation. The above research showed that the tilting deformation curve of a landslide accurately represents the deformation evolution process and is a crucial parameter for landslide warning and prediction.

Liu et al.^37^ conducted a model test study of a landslide's tilting deformation induced by rainfall and used its tilting deformation curve to predict its instability time. The model test of tilting deformation of locked-segment type landslides with retaining-walls is performed on this basis. This study conducts physical model tests to explore additional tilting deformation, failure characteristics of the locked segment, and the evolution mechanism of retaining-wall locked segment landslide under rainfall. Tilt sensors, micro earth pressure gauges, pore water pressure gauges, resistance strain gauges, and other instruments are installed to monitor the landslide evolution process. In addition, based on the tilting deformation curve of the landslides, an improved tangential angle approach and velocity reciprocal method are proposed for predicting landslide instability.

## Materials and methods

### Experimental materials

The geological conditions in the western Henan province are complicated. There is an increase in disasters and casualties across the region. Based on the analysis of topography, geomorphology, and other geological conditions in western Henan, the landslide slope in this area is between 30° and 45°^38^. The physical model test of retaining-wall locked segment landslide with a slope of 45° was carried out in this study to reflect in-situ conditions.

The size of the model box was 1.6 m × 0.5 m × 0.6 m (length × width × height). The test model consisted of bedrock, sliding body, and retaining-wall locked segment. The bedrock was made of masonry blocks and mortar. The landslide body material was silt from western Henan, which was dried and sieved. It was then used to fill the box to 0.4 m. Physical and mechanical parameters of the landslide body material, such as cohesion, internal friction angle, and permeability coefficient, were obtained through particle sieving, permeability, and direct shear tests, as shown in Table 1.

**Table 1:** Physical and mechanical properties of model material.

Material usage	Material	Dry density (g/cm^3^)	Moisture content	Cohesive force (kPa)	Internal friction angle (°)	Permeability coefficient (cm/s)
Model material	Silt	1.52	16	8	31	3.5 × 10^−5^

Relevant studies have shown that the locked segment is of low brittleness^39^. Based upon the existing studies of locked-segment type landslides^40,41^, water, gypsum, and sand were selected to simulate the retaining-wall type locked segment with a mass ratio of 1:1.4:1. According to the same ratio of the cylindrical sample, using YAW6206 electro-hydraulic servo press to carry out a uniaxial compression test, the uniaxial compressive strength was 3.27 MPa. The size of the locked segment was (length × height × thickness) 0.5 m × 0.42 m × 0.02 m, and a layer of paraffin was coated on the surface of the locked segment to ensure that its strength was not affected by rainfall infiltration.

### Experimental measurements

The rainfall device is the NLJY-10 portable artificial rainfall system from Nanlin Electronics., LTD. It consists of 27 nozzles of different sizes. The height of the rainfall device is 4 m, and its effective rainfall area is 4 × 4 m. The rainfall intensity is controlled by different pressure and nozzles; its variation range is 10–240 mm/h.

The monitoring equipment used low-cost and high-precision HCA726S biaxial tilt sensors, miniature earth pressure sensors, pore water pressure sensors, strain gauges, high-definition cameras, etc. Tilt sensors were integrated with a high-tech MEMS module. Volume was 56 mm × 46 mm × 20.5 mm, the range was ± 10°, with an accuracy of ± 0.008°, and the resolution was 0.0001°. The data acquisition frequency was set to 15 Hz. The micro earth pressure sensors and pore water pressure sensors were DMTY type gauges. The parameters are shown in Table 2.

**Table 2:** Sensor parameters.

Type of sensor	Model	Range ( kPa)	Size	Precision	Sensitivity coefficient
Earth pressure sensor	DMTY	0 to 20	φ20*6.5 mm	≤ 0.5%F·S	2.0
Pore-water pressure sensor	DMTY	− 90 to 10	Φ19*21 mm	≤ 0.5%F·S	2.0

**Figure 1:**
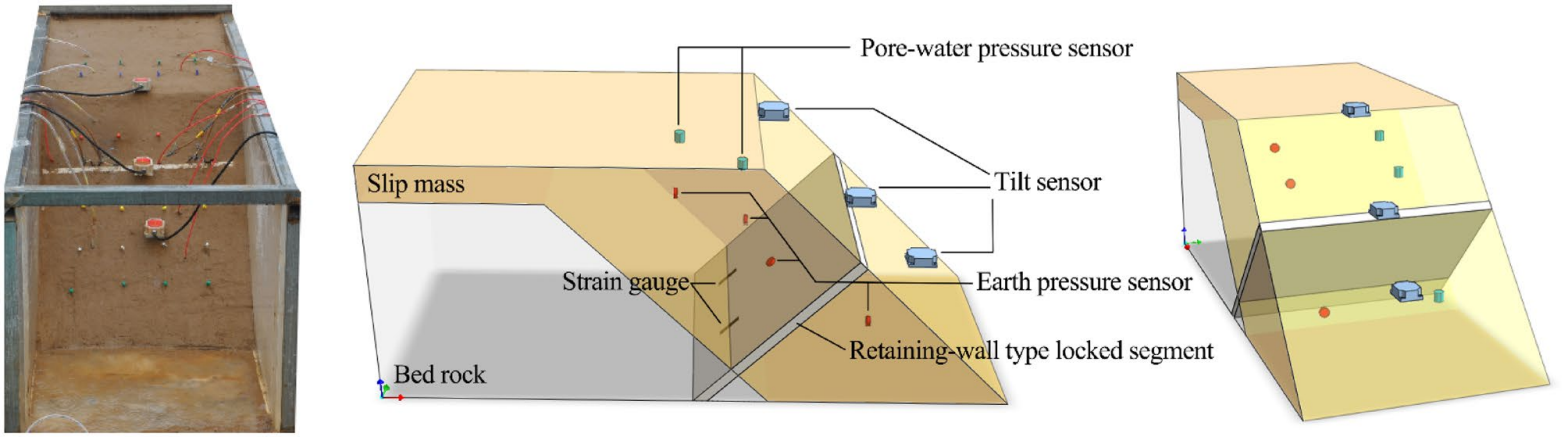
Landslide model and instruments layout.

In the model test, the tilt sensors T1–T3 were fixed to 120 mm long right-angle iron plates and inserted into the landslide to monitor the tilting deformation in the main sliding direction. Figure 1 was drawn using GTS NX (http://www.midasGTSNX.com) and Adobe Photoshop CC 2020 (Adobe Photoshop (cvbty.cn)). T1 was installed on the trailing edge of the landslide. T2 was fixed on the locked segment. T3 was installed on the front edge of the landslide. Pore-water pressure gauges P1, P2, and P3, and micro earth pressure gauges S1, S2, and S3 were buried on the trailing edge, the middle, and the front edge of the landslide, respectively, to a depth of 100 mm. They were used to monitor the landslide body stress throughout its evolution. The soil pressure gauge S4 was fixed on the locked segment closed to the trailing edge to monitor the change of earth pressure near the locked segment. A waterproof resistive strain gauge, model BF120-20AA-X30, with a sensitivity coefficient of 2.0%, resistance value of 120.0 Ω, and dynamic collection frequency of 500 Hz, was arranged on the locked segment around the bedrock contact surface.

During the test, a high-definition camera was used to take pictures on the slope surface every 5 min, and the dynamic evolution process was obtained by real-time video recording on the landslide side.

Based on the perennial average rainfall in western Henan, the rainfall intensity was set as 70 mm/h, and the rainfall duration was ~ 7000 s.

## Results and analysis

### Landslides deformation and stress response

**Figure 2:**
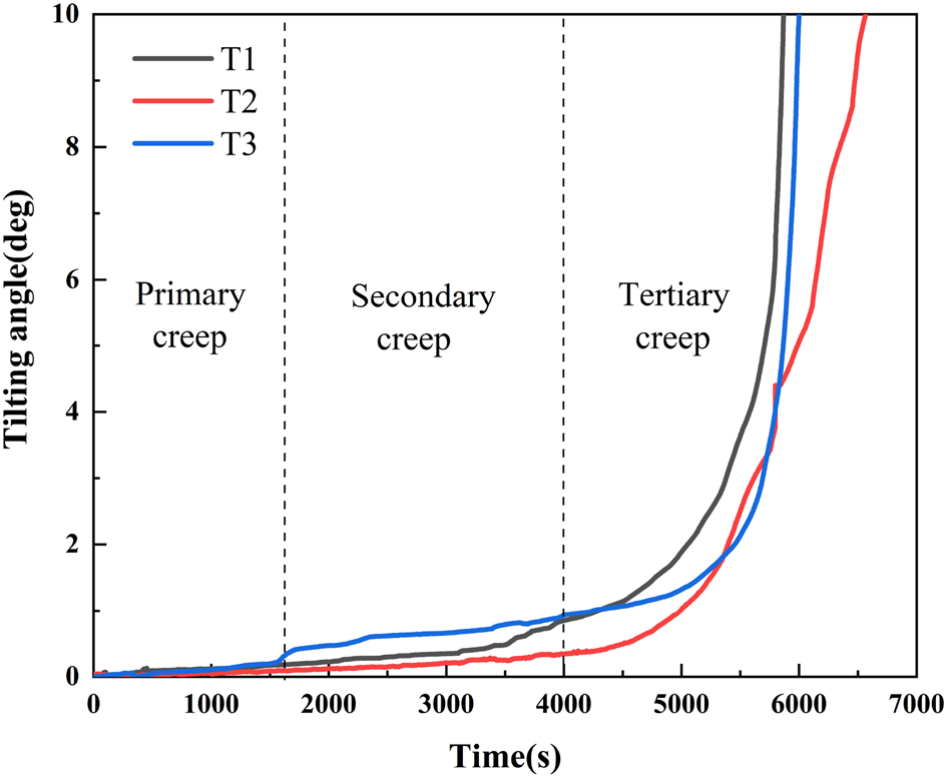
Tilting deformation curve of landslide.

Tilt sensors were used to monitor the tilting deformation of a landslide under a rainfall intensity of 70 mm/h. As shown in Fig. 2, the angle–time curve of the landslide is consistent with the displacement–time curve, with typical nonlinear characteristics, which can be summarized as primary creep, secondary creep, and tertiary creep stages^42^. The landslide's tilting deformation curve is almost stable in the initial rainfall stage. The tilting angle changes slightly, and the landslide is in the primary creep stage. As the rainfall continues to 1800s, the tilting deformation of the landslide front monitored by T3 begins to increase slowly. The monitoring curves of T1 and T2 also slowly and linearly increase. The landslide enters the secondary creep stage. When the rainfall lasts to about 4000 s, the tilting deformation curve of the landslide increases significantly. At this point, the tilting rate also slowly increases. The landslide then enters the tertiary creep stage, and then the tilting deformation curve of the landslide steeply increases until the sensors reach the range and fail.

**Figure 3:**
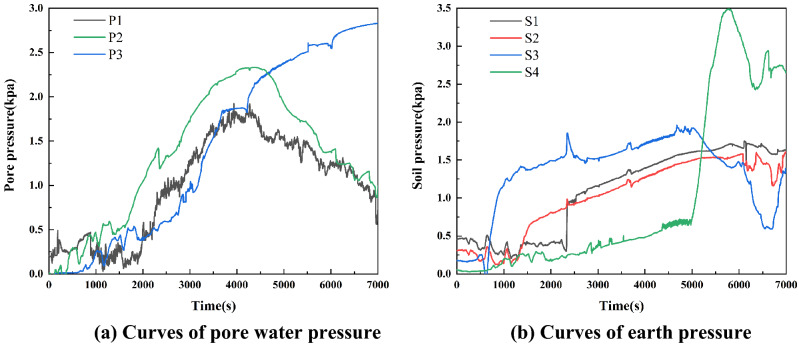
Stress response process of a landslide.

With rainfall infiltration, the pore water pressure and soil pressure change with the evolution of the landslide, which can further reflect the internal stress adjustment process. The pore water pressure at the trailing edge, middle edge, and front edge of a landslide was monitored by P1, P2, and P3; S1, S2, and S3 monitored the change of earth pressure at the trailing edge, middle part, and the front edge of landslide, respectively. S4 monitored the variation trend of earth pressure near the locked segment. As shown in Fig. 3, the change process of pore water pressure and earth pressure in a landslide body can be roughly divided into three stages: slow growth, rapid growth, and rapid decline.

At the beginning of rainfall, the landslide is in the primary creep stage. Due to gas in the pores, no stable seepage channel is formed in the slope body, and pore water pressure increases slowly. As the rainfall continues to ~ 1800 s, infiltration increases, and the landslide enters the secondary creep stage. Furthermore, the pore water pressure increases rapidly, and the soil mass changes from an unsaturated state to a saturated state. When the rainfall continues to ~ 4000 s, the landslide enters the tertiary creep stage. The effective stress of the slope body decreases. The landslide begins to slide, pore water is released, and P1 and P2 decreased significantly. Throughout the rainfall period, P3 continued to increase and remained at a high value due to the collapse of the landslide front and the contact between the pore water pressure gauge and water.

Rainfall infiltration causes soil to transition from an unsaturated state to a saturated state. At the beginning of rainfall, the increase of soil water content leads to an increase in sliding weight and a slow increase of earth pressure. As the rainfall continues, the landslide front is affected by slope runoff and rainfall erosion, and S3 is the first to show rapid growth. Then the soil pressure increases successively in the middle and back of the landslide due to the extrusion of the landslide's trailing edge. The earth pressure at the locked-in segment S4 increases rapidly when the rainfall lasts to 5000 s. It reaches its peak and decreases rapidly at about 5800 s when the external force on the locked segment exceeds its strength and fracture occurs. After the failure of the locked segment, accelerated failure of the landslide occurs. The earth pressure at gauges S2 and S3 along the trailing edge and the middle of the landslide begin to decrease.

For the locked-segment type landslide with a retaining-wall, the landslide evolution process under the condition of rainfall has a specific sequence of events. First, the landslide's front edge collapses. Next, the trailing edge is pushed along. Finally, the retaining-wall type locked segment is cut off, and the whole landslide undergoes instability failure.

**Figure 4:**
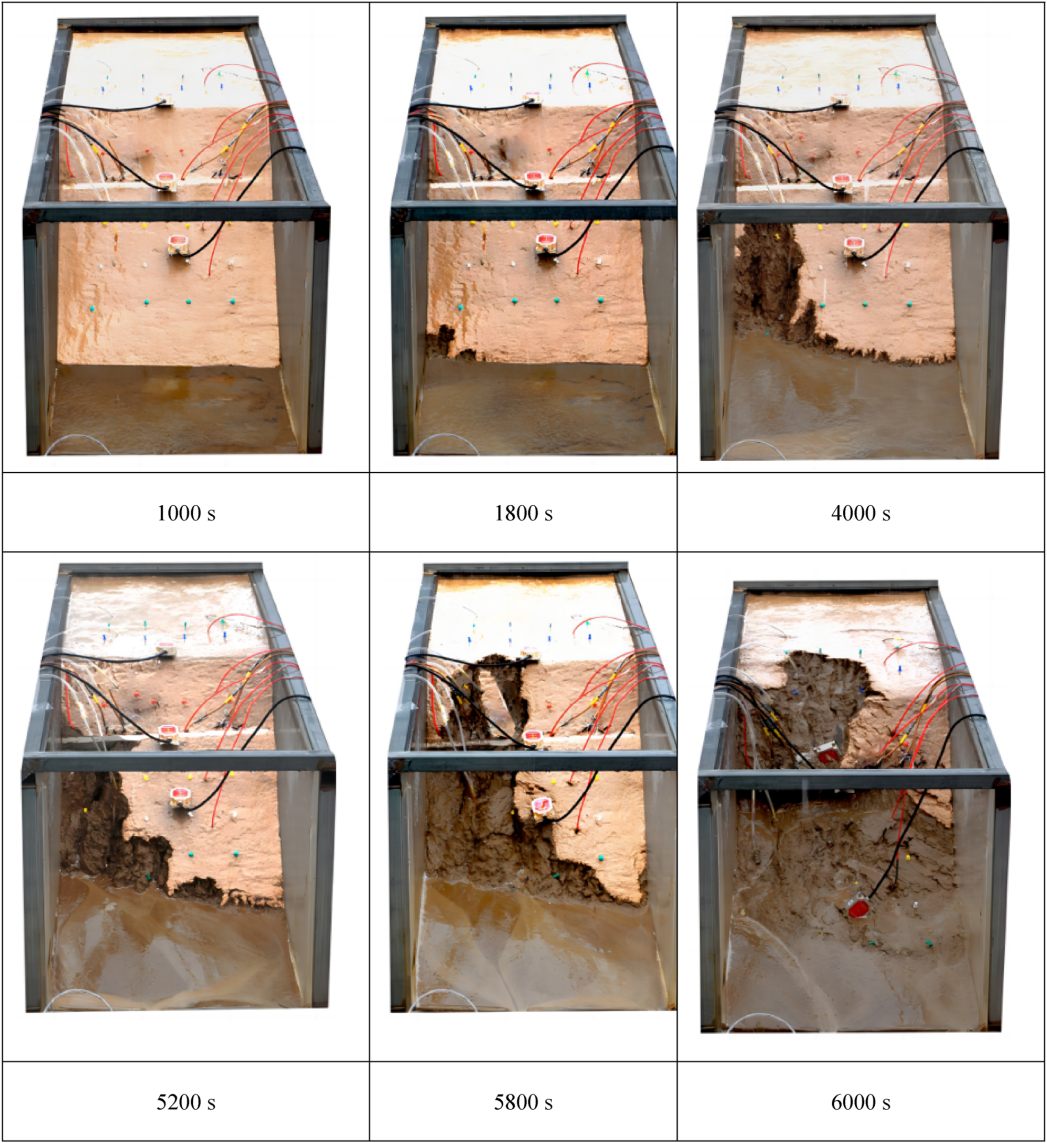
Evolution of landslide with retaining-wall locked segment.

As shown in Fig. 4, which processed by Adobe Photoshop CC 2020 (Adobe Photoshop (cvbty.cn)), the landslide is in the primary creep stage at the beginning of rainfall, and the overall tilting deformation is small. As the rainfall continues to 1800s, the landslide enters the secondary creep stage. At this time, small cracks appear at the landslide's foot. Due to rain erosion and slope confluences, small gullies appear at the foot of the landslide and continue to pull backward. As the rainfall lasts to ~ 4000 s, the landslide enters the stage of tertiary creep, and partial collapse occurs on the front edge and gradually spreads to the trailing edge of the landslide. At 5200 s, the landslide collapsed front extends upward to the locked-in segment and stops—a sizeable alluvial fan forms in the landslide front. With continuous rainfall, local gullies appear on the slope surface, eroding and destroying. Due to the increasing extrusion of the trailing edge, the locked segment is cut off ~ 5800 s, and the mass slides as a whole very quickly. At ~ 6000 s, the trailing edge of the landslide begins to settle, and obvious transverse tensile cracks appear until the whole landslide completely fails.

### Deformation characteristics of a retaining-wall locked segment

The monitoring data of tilt sensors T1, T2, and T3 were further analyzed to investigate the tilting deformation laws. First, Eq. (1) is utilized to calculate the tilting rates of T1, T2, and T3:

1 $$ \left| {dv_{i} } \right| = \left( {\frac{{\left| {d\theta } \right|}}{{{\text{d}}t}}} \right)_{i} = \frac{{{\text{d}}\theta_{i} - {\text{d}}\theta_{i - 1} }}{{t_{i} - t_{i - 1} }} \quad (i = 1, \;2, \ldots , \;n) $$

where *t*_*i*_ and *t*_*i−*1_ are the time; ${\text{d}}\theta$ is tilting angle increment from $t_{i - 1}$ to *t*_*i*_; $dv_{i}$ or $\left( {\frac{{\left| {{\text{d}}\theta } \right|}}{{{\text{d}}t}}} \right)_{i}$ is tilting rate, that is angle change rate at $t_{i}$.

**Figure 5:**
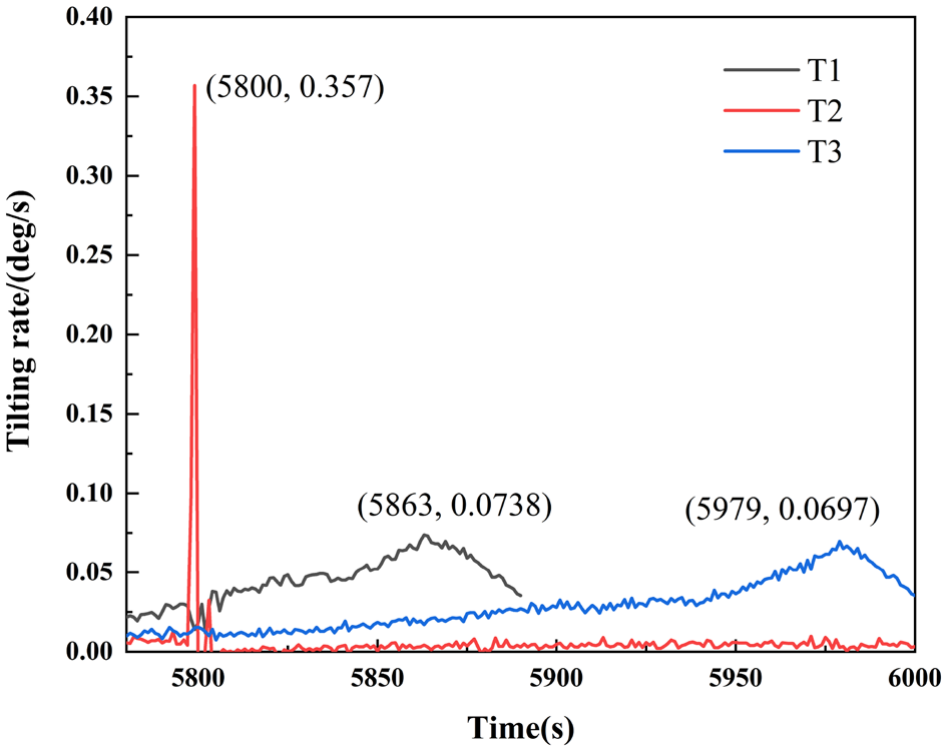
Variation curve of landslide tilting rate.

As shown in Fig. 5, the tilting rate of T2 suddenly increases to 0.357°/s at ~ 5800 s, while the tilting rate of T1 and T3 suddenly increases after 5800 s and is ~ 0.07°/s. This indicates that the locked segment fails around 5800 s. After the failure of the locked segment, the landslide becomes unstable and slides as a whole, and the tilting rate of its trailing edge and front edge increases suddenly. The results show that the locked segment is critical in controlling landslide stability.

Equation (2) was used to further calculate the tilting acceleration of T2.

2 $$ \left| {da_{i} } \right| = \left( {\frac{{\left| {dv} \right|}}{{{\text{d}}t}}} \right)_{i} = \frac{{{\text{d}}v_{i} - {\text{d}}v_{i - 1} }}{{t_{i} - t_{i - 1} }} \quad (i = 1,\;2, \ldots ,\;n) $$

where $t_{i}$ and $t_{i - 1}$ are the time (s); ${\text{d}}v$ is tilting rate increment from $t_{i - 1}$ to $t_{i}$ (X); $da_{i}$ or $\left( {\frac{{\left| {{\text{d}}v} \right|}}{{{\text{d}}t}}} \right)_{i}$ is tilting acceleration at $t_{i}$.

**Figure 6:**
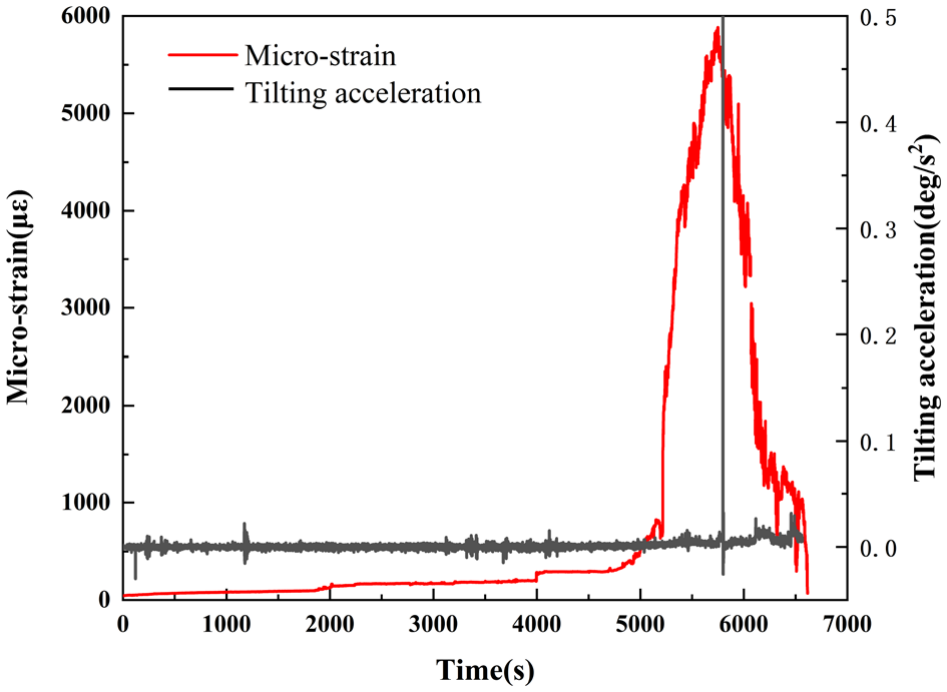
Failure characteristic curve of locked segment.

As shown in Fig. 6, the tilting acceleration of the locked segment is unchanged until ~ 5800 s, when a sudden increase occurs. This is consistent with the tilting rate change of the locked segment.

In addition, the monitoring data of the strain gauge installed in the locked segment were analyzed. Its variation trend is consistent with the tilting deformation characteristics previously reported. The strain in the locked segment is almost unchanged in the early rainfall period. At ~ 1800s, the landslide enters the secondary creep stage, and the strain increases slowly. At ~ 4000 s, the landslide enters the tertiary creep stage, and the strain rate increases significantly. The rainfall lasts until ~ 5200 s, and the strain of the locked segment increases steeply and reaches its peak at ~ 5800 s. This is consistent with the time when the tilting acceleration of the locked segment reaches the peak value, indicating that the locked segment breaks at this time and the strain gauge fails.

The analysis of the tilting rate, the tilting acceleration, and the strain of the locked segment show that the retaining-wall type locked segment has a particular controlling effect on the evolution of the landslide under rainfall and plays a decisive role in the overall stability of a landslide.

### Landslide forecast

The locked segment plays a crucial role in controlling the stability of the whole landslide, and the failure of the locked segment causes the landslide to accelerate in a short time. Therefore, the evolution process of the landslide can be further judged by analyzing the monitoring data of the tilt sensor T2 fixed on the retaining-wall type locked segment. This paper analyzed the tilting deformation curve of a locked-segment type landslide using tangential angle and reciprocal velocity methods. The instability criterion and warning information are put forward, proving that the tilt sensors can predict landslide instability.

#### Tangential angle method

Landslide prediction can be made according to the tangent angle of the displacement–time curve. Based on numerous landslide case studies, the tertiary creep stage of a landslide can be further divided into initial tertiary, medium tertiary, and high tertiary creep stages with tangent angles of 80° and 85° as the criterion^43^. Accordingly, the angle–time curve of the landslide was analyzed. To avoid the difference in the tangent angle of the curve caused by stretching or contraction of the coordinate axis, the coordinate axis of the T2 angle–time curve was transformed according to Eq. (3). The relationship between tilting angle and time in the primary creep stage and the tertiary creep stage is nonlinear, and it is constant in the secondary creep stage, so the tilting rate is used to convert the curve to the same time dimension of vertical and horizontal coordinates. That is,

3 $$ T(i) = \frac{\theta (i)}{v} $$

where $T\left( i \right)$ refers to the ordinate value of the same dimension as time after coordinate transformation; $\theta \left( i \right)$ refers to cumulative tilting angle; $v$ refers to the tilting rate at the secondary creep stage, and the tilting rate of T2 is 1.1 × 10^−4^°/s.

**Figure 7:**
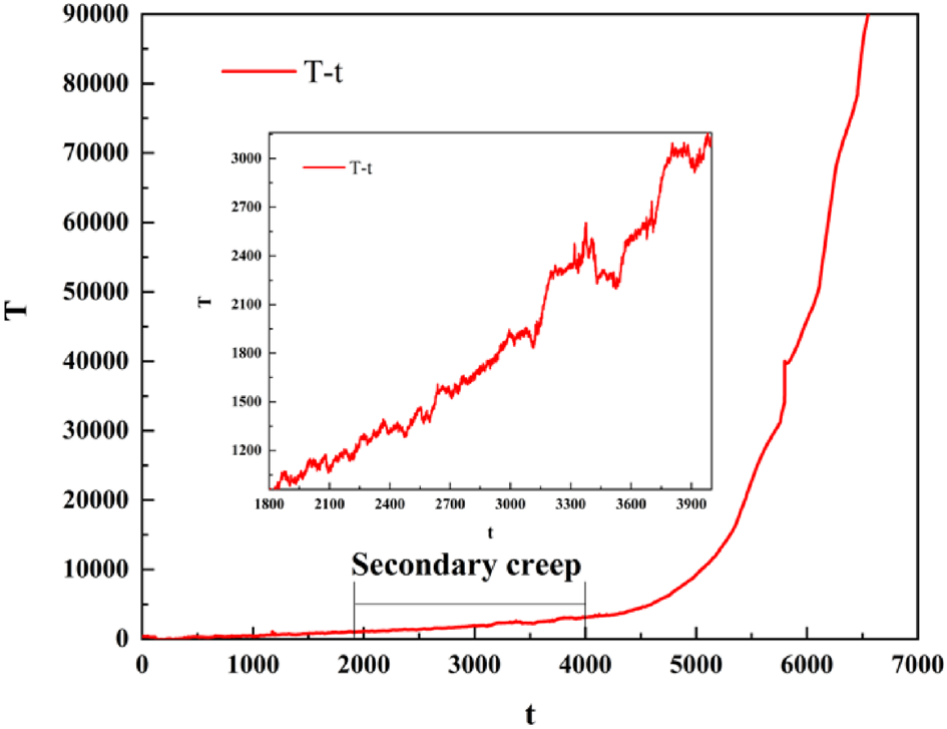
T–t curve after a coordinate transformation.

Figure 7 shows the T–t curve after a coordinate transformation. From 0 to 1800s, the tilting angle of the curve varies slightly. After 1800s, the tilting rate of the T–t curve linearly increases, and the landslide enters the secondary creep stage. By intercepting the curve at this stage and setting the same unit value for the vertical and horizontal coordinates, the tangent angle in this stage is 45°. At ~ 3980 s, the T/t values are all 1. After 3980 s, T/t continues to increase. Therefore, this time can be regarded as the starting time of the tertiary creep of the landslide, which is consistent with the termination time of the secondary creep stage of the landslide at 4000 s; that is, the termination time of the secondary creep of the landslide is 4000 s. After 4000 s, the change rate of the T–t curve increases and the angle exceeds 45°, and the landslide enters the tertiary creep stage.

**Figure 8:**
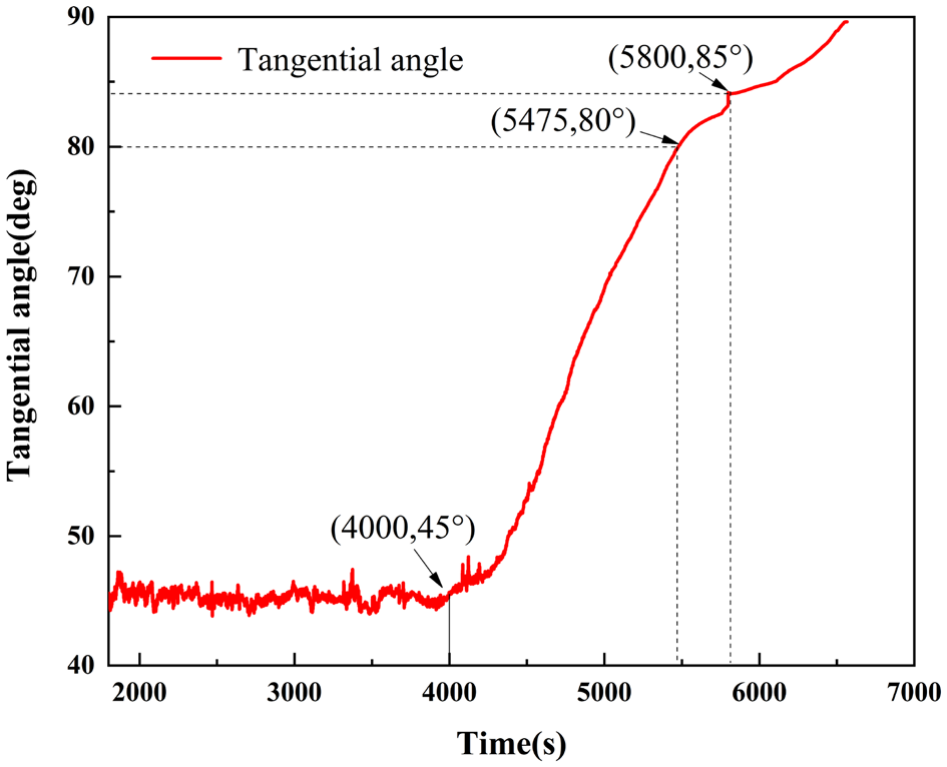
Curve of the tangential angle of T–t curve.

Equation (4) can be used to further calculate the value of the improved tangent angle of the T–t curve, and calculate the time curve of the tangent angle (Fig. 8).

4 $$ {\text{a}} = \arctan \frac{T(i) - T(i - 1)}{{t_{i} - t_{i - 1} }} = \frac{\Delta T}{{\Delta t}} $$

where a is the improved tangent angle; $t_{i}$, $t_{i - 1}$ is the monitoring time.

**Figure 9:**
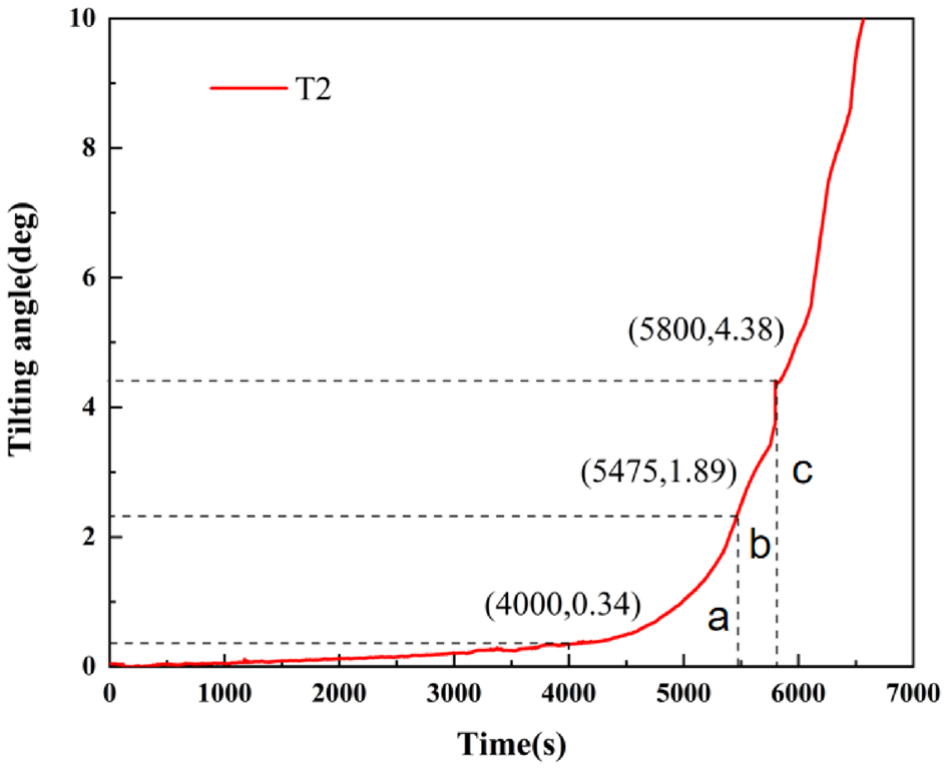
Curve of tertiary creep stage division, with (**a**) initial tertiary, (**b**) medium tertiary, and (**c**) high tertiary creep stages labeled on the diagram.

In the primary creep stage, the tangent angle of the T–t curve is < 45°. During the secondary creep stage, the tangent angle is ~ 45°. After the landslide enters the tertiary creep stage, the tangent angle is much greater than 45°. According to relevant studies and the summary of landslide cases^41^, when the tangent angle of the landslide is > 80°, the landslide deformation is significantly accelerated. When the tangent angle of the landslide is > 85°, the landslide shows signs of an impending slide, the deformation rate sharply increases, and the landslide becomes unstable and slides in the near future. Therefore, according to the improved tangent angle, the tertiary creep stage of a landslide can be further divided into initial tertiary, medium tertiary, and high tertiary creep stages, which correspond to the angle–time curve of a landslide (Fig. 9).

According to the T–t curve and the tangent angle of the curve after coordinate transformation of the retaining-wall locked segment landslide, the rainfall lasts ~ 4000 s and the tilting angle of the locked segment is 0.34°. The landslide enters the initial tertiary creep stage at this point. When the tangent angle is 80°, the landslide enters the medium tertiary creep stage. This occurs at about 5475 s of rainfall, corresponding to a tilting angle of the landslide of 1.89°; the tilting rate significantly increases. When the rainfall lasts until ~ 5800 s, the locked segment breaks, and the tilting angle is 4.38°. The landslide enters the high tertiary creep stage. Shortly after this point, the tilting angle curve rises sharply, and the landslide enters the state of impending slip.

Research shows that the landslide instability information can be qualitatively determined according to the improved tangent angle. When the tilting angle reaches 0.34°, the landslide enters the initial tertiary creep stage. When the tilting angle reaches 1.89°, the landslide begins the medium tertiary creep stage. When the tilting angle reaches 4.38°, the locked segment breaks and the landslide enters the high tertiary creep stage.

#### Reciprocal method of velocity

The reciprocal velocity method is widely used to predict the landslide instability time^24,33^. In the early stages of development, the tilting deformation of the unlocked segment landslide was studied, and the reciprocal velocity method was used to predict the landslide instability time^35^. Similarly, the reciprocal velocity method is used to predict the instability time of landslides in the locked segment; that is, the failure time is predicted using the tilting deformation curve of the locked segment.

**Figure 10:**
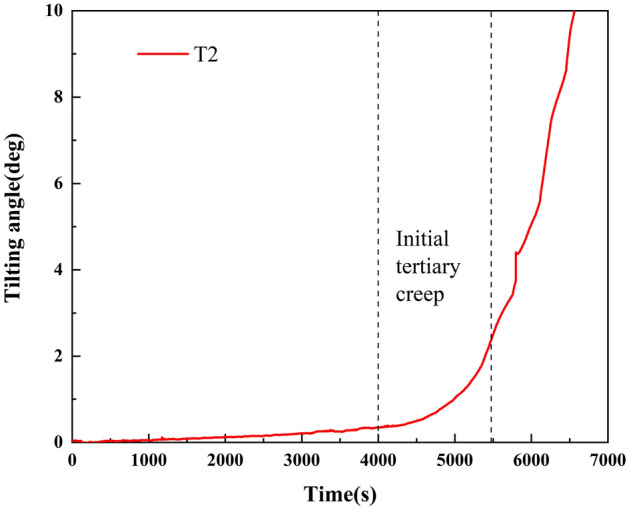
Tilting deformation curve of the retaining-wall locked segment landslide.

As shown in Fig. 10, when rainfall lasts for ~ 4000 s, the landslide enters the initial tertiary creep stage, and the tilting rate increases gradually. This stage is the critical stage for predicting landslide instability. This stage is analyzed, and an early warning system is proposed.

The tilting rate of the locked segment in the initial tertiary creep stage was calculated using Eq. (1).

Considering the influence of the monitoring data, the tertiary creep stage uses the data sequence of the angle change interval (0.2°). The linear relationship between the reciprocal of the tilting rate and time was determined using Eq. (5). The reciprocal of the tilting rate of 0 s/° (i.e., $\frac{{{\text{d}}t}}{{\left| {d\theta } \right|}} = 0$) was taken, getting the break time of the locked segment.

5 $$ \frac{dt}{{\left| {d\theta } \right|}} = \frac{ - t}{B} + \frac{{t_{f} }}{B} $$

where $$\frac{dt}{{\left| {d\theta } \right|}} $$ is the reciprocal tilting rate, B is the angle coefficient obtained from the linear relationship, and $$ t_{f}$$ is the slope failure time when the reciprocal tilting rate is 0 s/° ($$\frac{{{\text{d}}t}}{{\left| {{\text{d}}\theta } \right|}} = 0$$).

**Figure 11:**
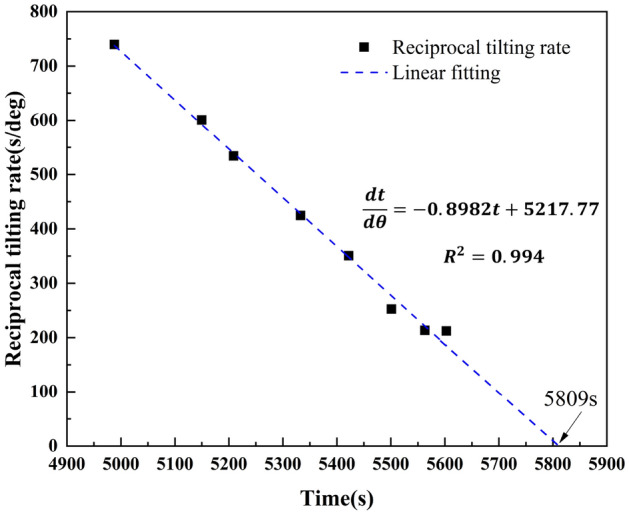
Curve of the reciprocal tilting rate over time of the retaining-wall locked segment landslide.

As shown in Fig. 11, the reciprocal ting rate was used to calculate the fracture time of the locked segment as 5809 s, which is consistent with the actual fracture time of the locked segment, 5800 s.

A landslide prediction model can be proposed by analyzing the angle–time curve. The tertiary creep stage of the curve can be divided into initial, medium, and high tertiary creep stages using the tangential angle method. Then, the tilting angle of the landslide in each stage can be qualitatively analyzed, and landslide prediction information can be suggested. The failure time of the locked segment can be predicted using the reciprocal velocity method according to the initial tertiary creep stage, that is, the instability time of a landslide.

## Discussion

This study uses tilt sensors to monitor the evolution process of retaining-wall locked segment landslides and analyze landslides' tilting and internal stress response characteristics under rainfall conditions. The results showed that the angle–time curve of a locked segment landslide with a retaining-wall has typical nonlinear evolution characteristics under rainfall conditions. Based on the locked segment's tilting acceleration and strain curves, the locked segment plays a crucial role in controlling the stability of landslides. The landslide instability criterion and prediction information are suggested according to its tilting deformation characteristics.

In recent years, tilt sensors have been widely used in landslide monitoring and prediction, and the tilting deformation of a landslide has become an important symbol of landslide instability.

Wang et al.^44^ determined that the tangential angle of displacements–time curve before landslide instability was generally 89°–89.5° through statistical analysis, which was used as one of the early warning and prediction criteria of landslides. They proposed the displacement tangential angle criterion, and the value of tangential displacement angle was further determined through coordinate axis transformation^41^. The angle–time curve of a landslide is consistent with the displacement–time curve, and both have typical creep characteristics that can be divided into three stages. Therefore, the same method is used to improve the angle–time curve of a landslide.

The improved displacement tangential angles of 80° and 85° are used as the segmentation points of the accelerated creep stage of a landslide, which can be divided into initial tertiary, medium tertiary, and high tertiary creep stages. The tilting angles of the landslide are 0.34°, 1.89°, and 4.38°, respectively, when the landslide enters the creep stage of initial tertiary, medium tertiary, and high tertiary. Similar to the displacement tangential angle method, the tilting tangential angle method is applied to suggest the landslide instability criterion, providing a new direction for monitoring and predicting landslide tilting deformation.

Xie et al.^33^ proposed a new landslide prediction method based on the tilting deformation characteristics of landslides. The linear relationship between the reciprocal of the tilting rate and time is utilized to predict the instability time of landslides. Liu et al.^35^ studied the tilting deformation characteristics of landslides under rainfall conditions and predicted the initial failure time and complete failure time according to the secondary creep and tertiary creep stages of angle–time curves. The landslide prediction information is proposed based on the tilting deformation curve, proving the effectiveness of monitoring landslide instability and landslide prediction using tilting sensors.

At present, almost all studies on the tilting deformation of landslides focus on non-locked segment landslides. In this paper, physical models of retaining-wall locked type landslides were carried out to study the tilting deformation and failure characteristics of the locked segment in the landslide evolution process and verify the critical role of the locked segment in controlling stability. In addition, two prediction methods (tangential angle and reciprocal velocity) are proposed for the tilting deformation curves of a landslide. These two methods have been widely used in landslide prediction.

In the paper, a landslide prediction method is proposed by using the tilting deformation curve of the retaining-wall locked segment, and landslide instability information is obtained by using the tangential angle of the improved angle–time curve, which provides a new method for landslide prediction. The failure time of the locked segment was predicted by the linear relationship between the tilting rate's reciprocal and the locked segment's time. The failure time of the locked segment was taken as the instability time of the landslide. Overall, the predicted instability time was consistent with the actual instability time.

## Conclusion

This study investigates the landslides' tilting deformation and stress characteristics in the evolution process under rainfall conditions using physical model tests of locked-segment type landslides with a retaining-wall. The locked-segment type landslide failure process is summarized, and the prediction information is presented using the angle–time curve. The main conclusions are as follows:

1. In the failure process of the locked segment, the strain, tilting acceleration, and earth pressure of the locked segment all peaked within 5800 s, which can be used as the criterion of the failure of the locked segment. According to the curve of the tilting rate of a landslide, the tilting rate of the trailing edge and front edge increases significantly after the locking segment fracture. Therefore, the locked segment plays a decisive role in controlling the stability of the landslide and further proves that the failure criterion of the locked segment can be proposed according to the monitoring data of the tilt sensors.
2. Based on the improved tangential angle method, the tertiary creep stage of a landslide can be divided into initial, medium, and high tertiary creep stages. The results show that when the tilting angle is 0.34°, the landslide enters the initial tertiary creep stage, and preventive measures should be implemented. When the tilting angle is 1.89°, the landslide enters the stage of medium tertiary creep, and an early warning should be given. When the tilting angle exceeds 4.38°, the landslide enters the stage of high tertiary creep and has significant characteristics of an impending slip state. It provides a criterion for landslide warnings and forecasts.
3. Based on the initial tertiary creep stage of the tilting deformation curve of the locked segment, the linear relation between the reciprocal of the tilting rate and time was proposed, and the corresponding linear equation was created. The failure time of the locked segment was predicted to be 5809 s using the model, which was consistent with the fracture time of the landslide locked segment (~ 5800 s). It shows that the method has universality and feasibility in a landslide early warning and prediction.

## Supplementary Information

Supplementary Information.

## Data Availability

The datasets used and/or analysed during the current study available from the corresponding author on reasonable request. And all data generated or analysed during this study are included in this published article [and its supplementary information files].
